# The EPIC-InterAct Study: A Study of the Interplay between Genetic and Lifestyle Behavioral Factors on the Risk of Type 2 Diabetes in European Populations

**DOI:** 10.1007/s13668-014-0098-y

**Published:** 2014-10-11

**Authors:** Nita G. Forouhi, Nicholas J. Wareham

**Affiliations:** Medical Research Council Epidemiology Unit, University of Cambridge School of Clinical Medicine, Institute of Metabolic Science, Cambridge Biomedical Campus, Cambridge, CB2 0QQ UK

**Keywords:** Type 2 diabetes, Prevalence, Diet, Physical activity, Nutritional biomarker, Aetiology, Prevention, Genetic factors, Gene-lifestyle interaction

## Abstract

The rising prevalence of type 2 diabetes around the world and the global pattern of variation in risk between countries have been widely attributed to an interplay between rising rates of obesity and poor lifestyles, and genetic or developmental susceptibility to disease. Although this general hypothesis has been in existence for more than 50 years, the precise mechanisms that may explain it have remained uncertain. Advances in technology and the application of new methods in large scale population studies have made it possible to study these mechanisms. The InterAct project, funded by the European Commission, is a large case-cohort study which has verified 12,403 incident cases of type 2 diabetes, facilitating the study of genetic and lifestyle factors on the risk of type 2 diabetes among European populations.

## Introduction

Type 2 diabetes is a major contemporary public health issue because of its high and rising prevalence worldwide and its association with diverse complications that lead to premature morbidity and mortality. The cost of the disease to individuals, carers, health systems, and society is considerable. The latest estimates from the International Diabetes Federation suggest that 382 million people had diabetes in 2013, and that this prevalence is projected to increase to 592 million by 2035 [1•].

Several randomised controlled trials have provided strong evidence that the primary prevention of type 2 diabetes is possible through lifestyle interventions aimed at changing diet and physical activity [2–6]. However, many uncertainties remain about how to translate this potential into action in real-world settings. Firstly, it is unclear how to determine the appropriate balance between investment in approaches to prevention that focus on individuals at high risk of diabetes as opposed to efforts that seek to impact on the entire population. Secondly, where high risk approaches are proposed, it is unclear whether detailed assessment of individual susceptibility to diabetes through estimation of genetic risk, for example, plays a role in identifying people who may benefit from individualised prevention. Finally there are uncertainties about the details of the nutritional and dietary guidelines that should form the basis of public health targets as opposed to recommendations for change among individuals at high risk. When the World Health Organization reviewed the evidence for the association between diet and the development of type 2 diabetes a decade ago, no dietary factor fulfilled the criteria to qualify for convincing grade evidence [7]. Progress has been made since then with evidence arising predominantly from US-based cohorts, but systematic, large-scale evidence from Europe-based populations was still unavailable. Moreover, the investigation of an interaction between genetic and lifestyle factors has been a challenge as this would require an adequately powered study with a large sample size, availability of genetic data as well as robustly collected valid data on lifestyle factors.

To address these research gaps, the InterAct project (http://www.inter-act.eu) was funded by the European Union (EU) within its FP6 framework to investigate the interaction of genes and lifestyle on the risk of type 2 diabetes. A feature of this study that made it suitable for this purpose was that it was embedded in a multi-country cohort study (The EPIC study) with heterogeneity of dietary exposures which were assessed within a standardised framework. The initial study was very large and with sufficient elapsed time so that InterAct was able to accrue four million person-years of follow-up and ascertain and verify 12,403 incident cases of type 2 diabetes. Previous studies on gene-lifestyle interaction have demonstrated the importance of correctly parameterising and characterising the main lifestyle exposures before proceeding to investigate interaction [8]. Therefore, a key initial stage in InterAct was the examination of the nature and magnitude of the relationship between a variety of lifestyle behavioural factors and incident diabetes. Not only did this process set the stage for the examination of interaction, but it is also of public health importance in its own right as it guides the design of public health interventions.

## The InterAct Project

The InterAct project is co-ordinated by the Medical Research Council Epidemiology Unit, Cambridge, and it is largely centred on constructing a nested case-cohort study within eight of the ten participating countries of the European Prospective Investigation into Cancer (EPIC) cohort study: France, Italy, Spain, UK, the Netherlands, Germany, Sweden, and Denmark. Collaborators in 26 European research centres ascertained and verified 12,403 cases of incident type 2 diabetes from a total cohort of 340,234 people with four million person-years of follow-up, and randomly selected 16,835 subcohort participants from the full cohort [9••]. After exclusions, a representative subcohort of 16,154 individuals remained. A design feature of the case-cohort design is that due to the process of random selection, a number of future disease cases are also included within the subcohort. Thus, the total sample of EPIC-InterAct included 12,403 case participants with type 2 diabetes, and 16,154 subcohort participants, of whom 778 formed an overlap group between the case-group and the subcohort group as they had incident type 2 diabetes during follow-up. The case-cohort design should be thought of as a subcohort study with additional incident cases added rather than as a case-control study. The statistical analysis for the case-cohort design accounts for the small number of cases in the subcohort. Incident type 2 diabetes cases were ascertained up until 31 December 2007 through a review of several sources of evidence including self-report, linkage to primary care registers, secondary care registers, medication use, hospital admissions and mortality data. No diabetes cases were ascertained solely by self-report, but rather confirmatory evidence was sought for all cases with information about incident type 2 diabetes with fewer than two independent sources, including a review of individual medical records in some centres [9••].

## The Association Between Diet, Nutrition, and the Risk of Type 2 Diabetes

Detailed self-report assessments of food intake were made in each participating centre using validated country-specific dietary questionnaires, mostly food frequency questionnaires (FFQ). A bespoke European nutrient database was used [10]. Comprehensive approaches to analysis were taken, including adjustment for energy intake, statistical models that included the residual method where relevant, measurement error correction using calibration of FFQ data against the 24-h recall in a subset of the sample, and adjustment for a range of potential confounding factors including known diabetes risk factors, demographic, social, lifestyle and dietary factors.

### Food Groups and the Development of Type 2 Diabetes

The publication of three meta-analyses had indicated a positive association between a diet rich in red and processed meat consumption and the risk of type 2 diabetes [11–13], with a focus largely on US-based studies. Though individual studies investigating this had started emerging from Europe [14–16], there remained a lack of a systematic approach and adequate sample size. EPIC-InterAct reported that across eight European countries, for every 50-g increase in consumption of red meat and processed meat, there was a higher risk of future type 2 diabetes, with a hazard ratio (HR) of 1.08 [95 % confidence interval (CI) 1.03, 1.13] and 1.12 (95 %CI: 1.05, 1.19), respectively, independent of a comprehensive range of potential confounding factors [17].

In contrast to the more consistent findings for meat intake and diabetes risk, there were previously inconclusive findings for the association between the consumption of dairy products and diabetes risk, in particular for dairy sub-types [18]. There was a general paucity of such research in Europe, which was surprising given that Europe has the highest intake of dairy products in the world [19]. A previous analysis in the EPIC study had shown substantial variation in the amount of total and types of dairy products consumed across Europe, with high cheese consumption in France and high yoghurt consumption in Sweden and the Netherlands [20]. With its varying dairy products intake, the EPIC-InterAct study reported that intake of total dairy products, or of milk, was not related to future diabetes risk, but the consumption of combined fermented dairy products (yoghurt; cheese; thick fermented milk) was associated with a reduced risk of incident diabetes (HR 0.88, 95 % CI 0.78, 0.99) [21].

Evidence on the role of fish intake for diabetes risk has been conflicting, ranging from proposed beneficial [22] to harmful [23, 24] to null effects [25]. Findings from the EPIC-InterAct study have indicated that only sub-types of fish intake, rather than total fish intake, may be related to diabetes risk [26]. Further to the InterAct findings, evidence is accumulating that geographic location plays an important role in the nature of the association between fish intake and diabetes risk [25, 27•].

Fruit and vegetable intake is a recommended component of a healthy diet, but little evidence was available generally, and particularly from Europe, for a link between intake and diabetes risk [28]. We conducted a systematic review of previously published literature and then performed a meta-analysis along with new data from EPIC-InterAct. Our analysis of 179,956 participants and 19,123 incident type 2 diabetes cases reported that those consuming the highest fruit and vegetable intake had a 7 % (95 % CI: 0.87 – 1.00) lower risk of diabetes when compared with the lowest intake consumers, with a particular benefit for consumption of green leafy vegetables [29]. This work has provided more convincing evidence for the benefits of fruit and vegetable consumption for the prevention of diabetes.

### Beverages and the Development of Type 2 Diabetes

There has been increasing interest in associations between the consumption of sweetened beverages and the risk of developing diabetes, but data from Europe had been sparse, with only one European country (Finland) contributing to a systematic review and meta-analysis [30•]. EPIC-InterAct investigated associations between both sugar-sweetened beverage (SSB) and artificially sweetened beverage (ASB) intake and the development of incident type 2 diabetes [31•]. In adjusted analyses, EPIC-InterAct reported a multivariable-adjusted 22 % (95 % CI: 9 % to 38 %) higher incidence of type 2 diabetes for every one serving/day (336 g standard size can of drink) of greater habitual consumption of SSB. A previous meta-analysis was unable to distinguish associations with and without adjustment for obesity [32], but in EPIC-InterAct there was a persisting 18 % higher incidence of diabetes (95 % CI: 6 % to 32 %) even after adjustment for adiposity. Though there was a positive association between habitual ASB consumption and diabetes risk, the association became non-significant after accounting for baseline obesity (body mass index); HR of 1.11 (95 % CI: 0.95, 1.31) for every one serving greater habitual consumption of ASB, in adjusted analyses including adjustment for energy intake and body mass index [31•].

EPIC-InterAct also reported that greater tea consumption was associated with a lower risk of type 2 diabetes among Europeans. Individuals who reported drinking at least four cups of tea per day had a 16 % (95 % CI: 0.71 – 1.00) lower risk of developing type 2 diabetes compared to those who reported no tea consumption [33].

### Dietary Patterns and the Development of Type 2 Diabetes

There has been increasing recognition that people consume overall diets within certain patterns which are largely healthy or unhealthy [34]. Therefore, it is of interest to study the association of dietary patterns with health, in addition to the associations of individual food groups and foods. In the first large-scale study, EPIC-InterAct has reported that people who ate a dietary pattern in concordance with the principles of the Mediterranean diet had a reduced risk of developing diabetes [35]. The InterAct Consortium further extended its enquiry to include other predefined dietary patterns and also applied reduced rank regression (RRR) as a mixture of a hypothesis-oriented and an exploratory approach that is aimed at identifying food group combinations that explain a maximum of variation in (disease-related) response variables [36]. In EPIC-InterAct there was no association between the alternative Healthy Eating Index (aHEI) and the Dietary Approaches to Stop Hypertension (DASH) score and diabetes risk, but inverse associations were observed for three RRR-derived dietary pattern scores [37]. The study confirmed that adherence to specific dietary patterns, commonly characterised by high intake of fruits or vegetables and low intake of processed meat, sugar-sweetened beverages, and refined grains, may lower type 2 diabetes risk.

### Nutrients, Nutritional Compounds, and the Development of Type 2 Diabetes

Past research on the association between protein intake and risk of diabetes included the limitations of small sample size or cross-sectional evidence [38, 39], lack of investigation of type of protein (animal or plant origin) [40] or lack of association in obesity-adjusted analyses [41]. In EPIC-InterAct the incidence of type 2 diabetes was higher in those with the highest intake of total protein [per 10 g: HR: 1.06 (95 % CI: 1.02, 1.09), *p*-trend < 0.001] and animal protein [per 10 g: HR: 1.05 (1.02, 1.08), *p*-trend = 0.001], but in contrast, intake of plant protein was not associated with type 2 diabetes [per 10 g: HR: 1.04 (95 % CI: 0.93, 1.16), *p*-trend = 0.098] [42], after adjustment for known confounders. These findings are consistent with advice to limit diets high in dietary proteins, particularly from animal sources.

EPIC-InterAct also investigated carbohydrates intake, together with their glycaemic properties, as expressed by the glycaemic index (GI) and the glycaemic load (GL), and found no association with diabetes risk [43]. This is in contrast to findings from three meta-analyses of prospective, predominantly American studies, published prior to InterAct findings, that reported increased diabetes risk in the highest GI and GL categories, ranging from an increased risk of 16 % (GI) and 20 % (GL) to 58 % (both GI and GL) [44–46]. Further reports of a positive association between both GI and GL and risk of incident type 2 diabetes subsequent to the publication of findings from InterAct are also at variance with the null InterAct finding [47]. The investigation of GI values across different countries or even within the same population (such as one of the participating cohorts in InterAct) highlighted an important issue that an expansion of GI tables and systematic GI value assignment to foods is needed to improve the validity of GI values in cross-study comparisons. Analyses on the association between fibre intake and diabetes risk are complete in InterAct, and will be published combined with an updated systematic review and meta-analysis in the near future.

EPIC-InterAct found no association between dietary energy density of solid and semi-solid foods and incident type 2 diabetes [48]. This work highlighted the complexities of consensus on how energy density should be calculated, the potential role of under- or over-reporting, and the challenges of teasing out direct (on T2D) versus indirect (on obesity, weight gain) effects of dietary energy density, but concluded that energy density was likely to have only a small influence on the development of T2D even if these complexities were overcome.

There has been interest in the potential effects of flavonoids, a group of nutritional compounds of the polyphenol family that are contained in plant-based foods such as fruits, vegetables, nuts, legumes, cocoa, some cereals, and in tea, wine, and juice, but past evidence has been inconclusive for association with diabetes [49–54]. EPIC-InterAct reported that total flavonoid intake tended towards an inverse association with incident diabetes (HR 0.90, 0.77, 1.04, *p*-trend = 0.04), but higher and stronger significant inverse associations were found for flavonoid sub-classes [55]. In particular, the HR when comparing the top quintile versus the bottom quintile of intake of flavonols were 0.81 (0.69, 0.95), and flavanols 0.82 (0.68, 0.99), including flavan-3-monomers 0.73 (0.57, 0.93) demonstrated an association with reduced incident diabetes. Further inspection of individual flavonoids (beyond classes of flavonoids) highlighted significant inverse trends between intakes of all individual flavan-3-ol monomers and diabetes incidence [56]. Additionally, proanthocyanidin dimers and trimers, but not proanthocyanidins with a greater degree of polymerisation were inversely associated. Among the flavonol sub-class, myricetin (HR 0.77, 0.64, 0.93) was associated with a lower incidence of T2D. Our findings provided the first comprehensive examination of both flavonoid classes and individual flavonoids, using a comprehensive flavonoid food composition database that was developed using both the Phenol-Explorer (the U.K. Food Standards Agency) and the U.S. Department of Agriculture databases [55]. These findings provide meaningful contributions toward our understanding of how a dietary pattern rich in plant-based foods helps with the prevention of type 2 diabetes.

In the context of strong epidemiological evidence that circulating concentrations of 25-hydroxy vitamin D [25(OH)D] are related inversely to the development of type 2 diabetes [57], EPIC-InterAct also appraised the role of dietary vitamin D, as a contributing source of circulating 25(OH)D levels. The study reported a null association between vitamin D intake and risk of diabetes in both the total and sex-specific analyses. This should be interpreted in light of the relatively greater contribution of endogenous synthesis of 25(OH)D following sunlight exposure, and thus the evidence from our large-scale study confirming no association of type 2 diabetes with dietary vitamin D intake provides important information in helping to distinguish between dietary and endogenously synthesised vitamin D.

## Moving Beyond Self-Report Assessment of Diet to Using Nutritional Biomarkers

To overcome the well-acknowledged limitation of measurement error in nutritional epidemiology, InterAct has invested substantial effort into generating evidence on blood-based objective biomarkers that reflect nutritional intake.

There has been little comprehensive assessment of objectively measured circulating fatty acids in relation to diabetes risk across the range of individual fatty acids of varying carbon chain lengths. Reasons include the expense of measurement and the challenge of measuring them in large studies due to the substantial laboratory run-time and personnel time needed to process fatty acids on a large scale. In a collaboration between analytical chemists and epidemiologists, InterAct undertook to establish a high throughput method to measure 37 fatty acids in the plasma phospholipid fraction at the MRC Human Nutrition Research, Cambridge [58]. This enabled the measurement of plasma phospholipid fatty acids on an unprecedented scale, allowing the measurement of nearly 28,000 EPIC-InterAct samples.

**Fig. 1:**
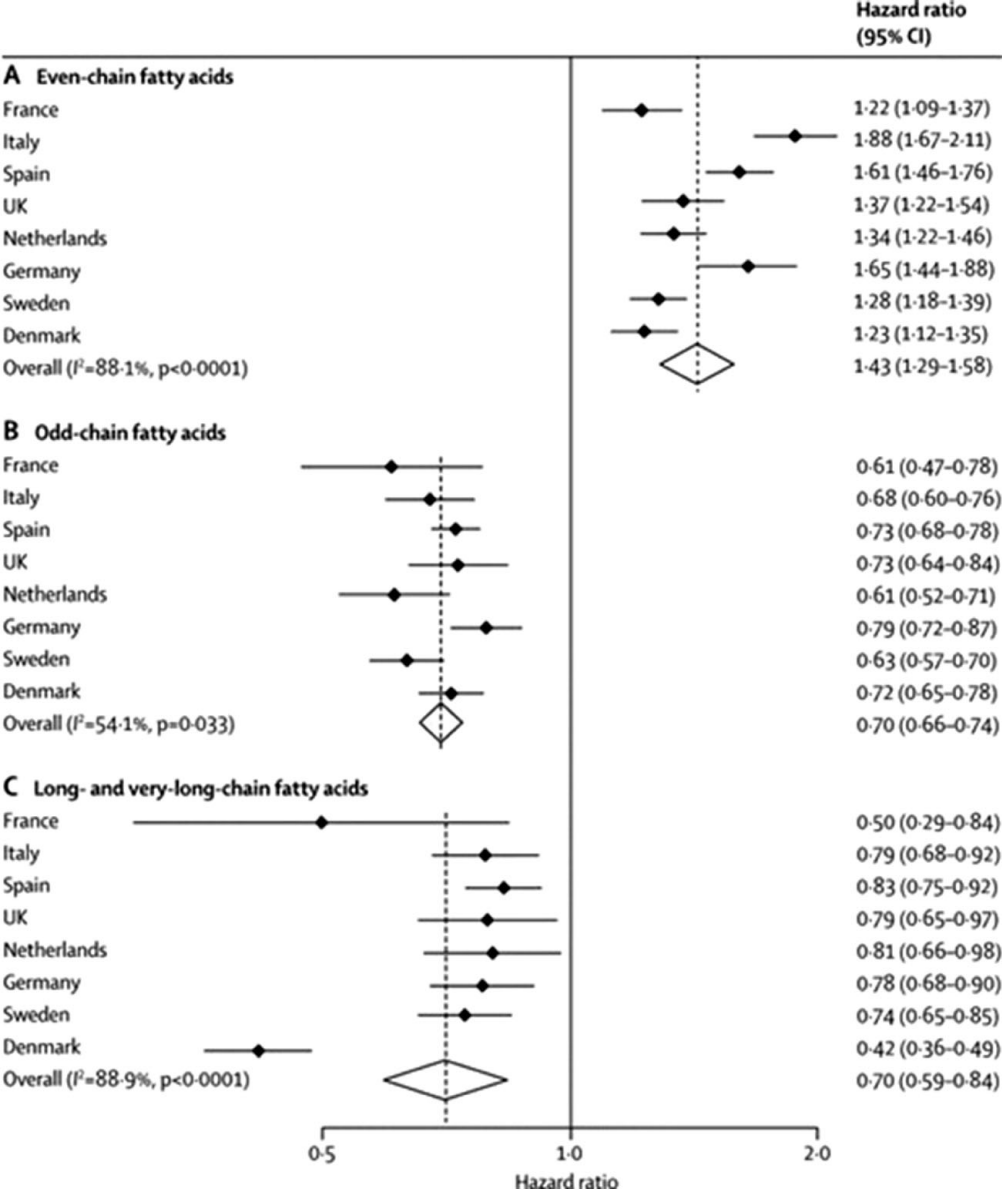
Hazard ratios and 95 % CIs for associations between plasma phospholipid saturated fatty acids and incident type 2 diabetes in EPIC-InterAct. Associations per 1 SD difference in (A) even-chain fatty acids (saturated fatty acid [SFA] group 1: the sum of 14:0, 16:0, and 18:0), (B) odd-chain fatty acids (SFA group 2: the sum of 15:0 and 17:0), and (C) long- and very-long-chain fatty acids (SFA group 3: the sum of 20:0, 22:0, 23:0, and 24:0) and type 2 diabetes. Estimates are per country and the pooled estimate is based on random-effects meta-analysis. The analyses included 12 132 cases of type 2 diabetes and 15 919 people in the subcohort (including 755 individuals with type 2 diabetes in the subcohort); used age as the underlying time variable; and were adjusted for centre, sex, smoking status, alcohol intake, physical activity, education level, total energy intake, and BMI. Reproduced from Forouhi NG, Koulman A, Sharp SJ, et al. Differences in the prospective association between individual plasma phospholipid saturated fatty acids and incident type 2 diabetes: the EPIC-InterAct case-cohort study. The Lancet Diabetes & Endocrinology. 2014; pii: S2213-8587(14)70146-9, with permission from Elsevier

In analyses focused on nine individual saturated fatty acids (SFAs), there were opposite directions of association with diabetes with SFA of varying carbon chain lengths [59•]. As shown in Fig. 1, odd-chain SFAs were associated inversely with incident diabetes [HR (95% CI) per 1 SD difference for 15:0 (pentadecanoic acid) 0.79 (0.73, 0.85), and for 17:0 (heptadecanoic acid) 0.67 (0.67, 0.71)], while even-chain SFAs were positively associated [14:0 (myristic acid) HR 1.15 (1.09, 1.22), 16:0 (palmitic acid) HR 1.26 (1.15,1.37), and 18:0 (stearic acid) 1.06 (1.00, 1.13)]. The very-long chain SFA (number of carbon atoms in chains varying between 20 and 24) were associated with lower diabetes incidence (HR ranging between 0.72 and 0.81 per 1 SD difference). The findings for odd-chain SFA can be understood in terms of their exogenous source from dairy fat [60–62] as well as the positive correlations in EPIC-InterAct between dairy products and the sum of pentadecanoic (15:0) and heptadecanoic (17:0) acids. This is in line with recent work, including from EPIC-InterAct, indicating that dairy products consumption is associated with lower risk of diabetes [18, 21, 63•]. The findings for even-chain SFA are more complex as these SFA appear to be mainly derived from hepatic endogenous synthesis (de novo lipogenesis), stimulated by intakes of carbohydrates and alcohol as shown in experimental studies [62, 64–67], and confirmed to some degree by InterAct findings: stronger correlations of even-chain SFA with intakes of potatoes, soft drinks, and alcohol than with direct dietary sources such as meat or dairy. Very little is currently known about the very-long chain SFA, and EPIC-InterAct provides impetus for further investigation of this group of SFA.

Taken together, EPIC-InterAct findings indicate that SFAs are not a single homogenous group, and recognise differences between the differential health effects of subtypes of blood SFAs. The implication of this research is that it recognises that it may not be enough to provide public health messages about overall saturated fat intake, but that more nuanced messages acknowledging the food sources of different types of SFAs are required.

The biomarkers plasma vitamin C [68], 25-hydroxy vitamin D [57], and carotenoids are currently being measured in stored samples from EPIC-InterAct participants. The availability of a broad range of nutritional biomarkers in EPIC-InterAct will open up possibilities for more robust testing of causality of proposed diet-diabetes associations using genetic Mendelian randomisation experiments [69, 70].

## The Association Between Physical Activity and the Risk of Type 2 Diabetes

The physical activity questionnaire used in EPIC-Europe is relatively simple and includes questions that quantify physical activity at work and in recreation. Whilst originally it was intended that analysis could use the concepts of duration, intensity, and frequency of activities to provide a quantitative summary of total activity, initial research demonstrated that quantification was not valid [71]. This analysis also suggested that an ordered categorical variable that created a global index of physical activity for individuals without the attribution of specific estimates of energy expenditure was valid and repeatable. Subsequent work demonstrated that this index predicted other clinical endpoints such as cardiovascular disease, increasing its face validity. Within InterAct, a cross-country validation study was undertaken showing the validity of this four category summary index of activity in all 10 countries in EPIC [72]. In addition to demonstrating overall validity, this analysis also quantified the energy expenditure difference between physical activity categories, suggesting that each category difference was equivalent to a difference in physical activity energy expenditure of 380 – 460 kJ per day or 20 min of brisk walking. InterAct then went on to quantify the relationship between this summary index of activity and the incidence of diabetes, showing that for each category difference in the physical activity index, there was a 13 % reduction in risk of type 2 diabetes in men and 7 % in women [73•]. This quantification of the dose-response relationship is of value in guiding expectations about what can be achieved through small changes in population-level physical activity.

## Interaction Between Genes and Lifestyle Factors on the Risk of Type 2 Diabetes

Through the studies described above, InterAct has, therefore, robustly characterised the relationship between the main lifestyle exposures and diabetes, which not only guides public health interventions, but also provides a platform for investigating how those associations are modified by genetic and developmental susceptibility. In the first such analysis, InterAct examined the association of a series of proven candidate genes for type 2 diabetes with disease risk examining interaction with lifestyle factors [74•]. The effect of the genetic score was significantly greater in younger individuals (*p*-value for interaction = 1.20 × 10^-4^). Relative genetic risk (per standard deviation; 4.4 risk alleles) was also larger in participants who were leaner, both in terms of body mass index (*p*-value for interaction = 1.50 × 10^-3^) and waist circumference (*p*-value for interaction = 7.49 × 10^-9^). The analysis found no significant interactions between the genetic score, comprising these variants, and sex, diabetes family history, physical activity, or dietary habits assessed by a Mediterranean diet score. We concluded that the relative effect of this diabetes genetic risk score was greater in younger and leaner participants, but that because this sub-group is at low absolute risk, they would not be a logical target for preventive interventions. By contrast, we argued that the high absolute risk associated with obesity at any level of genetic risk highlighted the importance of universal rather than targeted approaches to lifestyle intervention. This conclusion is limited to the set of genetic variants that were examined in this particular analysis, and future work will focus on genomewide approaches and sets of genetic variants selected by specific biological hypotheses.

## Conclusions and Public Health Implications of the InterAct Study

Beyond the specific conclusions of individual analyses that are summarised in this paper, the overall importance of the findings from the InterAct study is that it is creating a body of literature based on a large scale cohort across different European countries that forms a valid and robust foundation on which public health recommendations can be formed. In some instances, smaller studies have previously generated conflicting results and the strength of InterAct lies in the robustness of its conclusions. The development of additional layers of information, including objective nutritional biomarkers and genetics has opened up multiple lines of enquiry including the investigation of interaction and detailed examination of causal inference using genetic variants as instrumental variables. Such studies will not prove or disprove whether specific nutritional factors are causally linked to type 2 diabetes, but they can provide additional information which goes beyond the traditional observational epidemiological approach which is inherently limited by confounding. Whilst some have argued that the problems of confounding are only addressable by randomised controlled trials, the costs of undertaking trials of different preventive interventions are so prohibitive that there needs to be an investment into alternative approaches that can inexpensively provide additional information about causal interpretation, and thus provide greater confidence to public health recommendations. The structure established within the InterAct study provides a platform for such investigations in the future.
